# The NADPH Oxidase Nox4 Controls Macrophage Polarization in an NF*κ*B-Dependent Manner

**DOI:** 10.1155/2019/3264858

**Published:** 2019-04-18

**Authors:** V. Helfinger, K. Palfi, A. Weigert, K. Schröder

**Affiliations:** ^1^Institute for Cardiovascular Physiology, Goethe-University, Frankfurt, Germany; ^2^Institute for Biochemistry I, Goethe-University, Frankfurt, Germany

## Abstract

The family of NADPH oxidases represents an important source of reactive oxygen species (ROS) within the cell. Nox4 is a special member of this family as it constitutively produces H_2_O_2_ and its loss promotes inflammation. A major cellular component of inflammation is the macrophage population, which can be divided into several subpopulations depending on their phenotype, with proinflammatory M(LPS+IFN*γ*) and wound-healing M(IL4+IL13) macrophages being extremes of the functional spectrum. Whether Nox4 is expressed in macrophages is discussed controversially. Here, we show that macrophages besides a high level of Nox2 indeed express Nox4. As Nox4 contributes to differentiation of many cells, we hypothesize that Nox4 plays a role in determining the polarization and the phenotype of macrophages. In bone marrow-derived monocytes, ex vivo treatment with LPS/IFN*γ* or IL4/IL13 results in polarization of the cells into M(LPS+IFN*γ*) or M(IL4+IL13) macrophages, respectively. In this ex vivo setting, Nox4 deficiency reduces M(IL4+IL13) polarization and forces M(LPS+IFN*γ*). Nox4-/- M(LPS+IFN*γ*)-polarized macrophages express more Nox2 and produce more superoxide anions than wild type M(LPS+IFN*γ*)-polarized macrophages. Mechanistically, Nox4 deficiency reduces STAT6 activation and promotes NF*κ*B activity, with the latter being responsible for the higher level of Nox2 in Nox4-deficient M(LPS+IFN*γ*)-polarized macrophages. According to those findings, *in vivo*, in a murine inflammation-driven fibrosarcoma model, Nox4 deficiency forces the expression of proinflammatory genes and cytokines, accompanied by an increase in the number of proinflammatory Ly6C^+^ macrophages in the tumors. Collectively, the data obtained in this study suggest an anti-inflammatory role for Nox4 in macrophages. Nox4 deficiency results in less M(IL4+IL13) polarization and suppression of NF*κ*B activity in monocytes.

## 1. Introduction

Reactive oxygen species (ROS) regulate a variety of complex cellular processes including angiogenesis, inflammation, differentiation, and proliferation. The family of NADPH oxidases (Nox) consists of 7 members with tissue- and cell type-specific expression profiles. The main function of all family members is a controlled ROS production [1]. Importantly, the NADPH oxidases differ in the type of ROS produced. While Nox2 upon activation produces ·O_2_^−^, Nox4 is constitutively active and predominantly produces H_2_O_2_ [2, 3].

Inflammation and wound healing are processes that strongly depend on the function of macrophages. Macrophages are quite heterogeneous and represent a group of diversely polarized cells from the same monocyte origin [4]. The nomenclature of polarized macrophages has been changed recently. In particular, the M1 and M2 phenotypes have now been replaced by M(LPS+IFN*γ*) and M(IL4+IL13), respectively, according to the stimulation by cytokines forcing in vitro polarization to one or the other phenotype [5]. We followed this new nomenclature throughout the manuscript.

Nox2 and its product ·O_2_^−^ promote an M(LPS+IFN*γ*) phenotype with phagocytic activity and proinflammatory properties [6, 7]. In contrast, in tissue remodeling and wound healing, M(IL4+IL13) polarization of macrophages is characterized by both reduced Nox2 activity and reduced superoxide anion production [8]. H_2_O_2_ is a second messenger that enforces the polarization of monocytes to the M(IL4+IL13) phenotype (despite a lower Nox2-dependent ROS production observed in other studies [9]). Although, there is evidence that Nox4 is expressed in macrophages [10], this is rather inconsistent throughout the literature, leading to the conclusion that Nox4 expression is dynamic over the course of a macrophage life. Nox4 is a major determinant of differentiation of a number of cells, including adipocytes [11] and osteoclasts [12]. Therefore, we hypothesize that Nox4 plays a role in macrophage polarization. With the aid of an *in vivo* model of tumorigenesis, as well as isolated murine bone marrow and human blood monocytes, we analyzed the contribution of Nox4 in macrophage polarization.

## 2. Material and Methods

### 2.1. Material

The following chemicals were used: 3-methylcholanthrene (MCA), NaCl, NH_4_Cl, NaHCO_3_ Hank's BSS without Ca^2+^ and Mg^2+^, Trypsin-EDTA solution (T3924) and LPS from Sigma-Aldrich (Munich, Germany), Dulbecco's PBS (Gibco, Life Technologies, Carlsbad, CA, USA), Hank's buffer, Sybr Green from Bio-Rad (California, USA), Tris (Carl Roth) NF*κ*B inhibitor #sc-3060 from Santa Cruz (Texas, USA), and GKT 137928 from Genkyotex (Switzerland). IL4, IL13, and IFN*γ* were purchased from PeproTech (NJ, USA). The following antibodies were used: anti-*β*-actin (AC-15) from Sigma-Aldrich (Munich, Germany), pSTAT6, STAT6, pSTAT1, and STAT1 from Cell Signaling (Danvers, MA, USA), and p65, *β*-tubulin, and topoisomerase from Santa Cruz (Texas, USA). YM1 was from Chemicon-Millipore (Darmstadt, Germany), and YY1 was from Bethyl Laboratories (Texas, USA).

### 2.2. Animals and Animal Procedures

All animal experiments were approved by the local governmental authorities (approval numbers: F28/27 and F28/46) and were performed in accordance with the animal protection guidelines. C57Bl/6J and Nox2y/- mice were purchased from Jackson Laboratories (Bar Harbor, Maine). Nox4-/- mice were generated by targeted deletion of the translation initiation site and of exons 1 and 2 of the Nox4 gene [13] and backcrossed into C57Bl/6J for more than 10 generations. Nox1y/- mice, kindly provided by Karl-Heinz Krause and previously characterized, were used for the same experiments [14]. Mice were housed in a specified pathogen-free facility with 12/12 hours day and night cycle and free access to water and chow. All experiments were performed with male mice at the age of 10-12 weeks. To induce fibrosarcomas, the chemical carcinogen MCA was injected subcutaneously into the right flank of the mice. In response to this, tumors were formed within the next three to four months. Once the tumors reached a diameter of 1.5 cm (around 100 days), mice were sacrificed by isoflurane anesthesia and subsequent decapitation. Subsequently, the tumor tissue was processed for biochemical analysis.

### 2.3. Cell Culture

Cell populations were isolated using the tumor dissociation kit for the mouse and the gentleMACS Dissociator (Miltenyi Biotec, Bergisch Gladbach, Germany), following the manufacturer instructions. Briefly, tumor tissue was homogenized enzymatically, erythrocytes were lysed, and only fibrosarcoma cells were cultured whereas the rest of the cell suspension was only used for FACS analysis. Murine monocytes were isolated from bone marrow by flushing the bones with PBS containing 1% of PenStrep. Cells were filtered (Falcon; #340605, BD) and centrifuged, and erythrocytes were lysed. Erythrocyte depletion buffer contained 155 mM NH_4_Cl, 10 nM NaHCO_3_, and 100 nM EDTA in double distilled water,pH = 7.4. For isolation of human monocytes, whole blood samples were centrifuged (400 ×g for 30 minutes) on a Ficoll gradient (Bicoll separation solution #L6115, Millipore) without brake. In order to force macrophage development, human peripheral blood mononuclear cells (PBMCs) and murine bone marrow-derived monocytes were cultured in Dulbecco's modified Eagle's medium (DMEM+glutaMAX) (Gibco, Life Technologies, Carlsbad, CA, USA), supplemented with 10% fetal calf serum (FCS), 1% penicillin (50 U/ml), and streptomycin (50 *μ*g/ml), as well as 20% conditioned medium of L929 cells (contains M-CSF) for one week. Media were changed every 4 days. Before polarization, medium was exchanged to an unsupplemented DMEM/FCS. Polarization to M(LPS+IFN*γ*) was induced by 1 *μ*g/ml LPS and 100 U/ml IFN*γ*; and M(IL4+IL13) polarization by IL4 and IL13 100 ng/ml each. After 4 hours, cells were used for nuclear extraction, Western Blot, PCR, or ROS measurements.

### 2.4. mRNA Isolation and RT-qPCR

Total mRNA from frozen homogenized tissue and isolated cells was obtained with an RNA-Mini-kit (Bio&Sell, Feucht, Germany) according to the manufacturers' protocol. Random hexamer primers (Promega, Madison, WI, USA) and SuperScript III Reverse Transcriptase (Invitrogen, Darmstadt, Germany) were used for cDNA synthesis. Semi-quantitative real-time PCR was performed with the Mx3500P qPCR cycler (Agilent Technologies, Santa Clara, CA, USA) using the PCR Sybr Green qPCR Mix with ROX (Bio&Sell, Feucht, Germany) and appropriate primers. Relative expression of target genes was normalized to eukaryotic translation elongation factor 2 (EF2), analyzed by the delta-delta-ct method. Primer sequences are listed in Table 1.

### 2.5. Protein and Western Blot Analysis

For protein isolation, cells were lysed in a buffer containing 20 mM Tris/cl pH 7.5, 150 mM NaCl, 10 mM NaPP_i_, 20 nM NaF, 1% Triton, 10 nM okadaic acid (OA), 2 mM orthovanadate (OV), protein inhibitor mix (PIM), and 40 *μ*g/ml phenylmethylsulfonylfluorid (PMSF). Separation of nucleus and cytosol was achieved by lysing the cells in hypotonic buffer (10 nM HEPES pH 7.9, 10 nM KCl, 0.1 mM EDTA, 0.1 mM EGTA, 1% Nonidet, 10 mM DTT, protein inhibitor mix (PIM), and 40 *μ*g/ml phenylmethylsulfonylfluorid (PMSF)). Cells were centrifuged at 17000 g, and the supernatant was collected as the cytosolic fraction. The pellet was further lysed with a hypertonic buffer (20 mM HEPES pH 7.9, 0.4 M NaCl, 1 mM EDTA, 1 mM EGTA, 10 mM DTT, protein inhibitor mix (PIM), and 40 *μ*g/ml phenylmethylsulfonylfluorid (PMSF)). After centrifugation at 17000 g, the supernatant contained most soluble nuclear proteins, while membranes, organelles, and DNA were collected in the pellet. Protein content was determined with the Bradford assay [15]. Samples were boiled in reducing the Laemmli sample buffer and were subjected to SDS-PAGE followed by Western Blotting. After incubation with first antibodies, membranes were analyzed with an infrared-based detection system, using fluorescent dye-conjugated secondary antibodies from LI-COR Biosciences.

### 2.6. Electrophoretic Mobility Shift Assay

The electrophoretic mobility shift assay (EMSA) was performed according to the manufacturer protocol (LI-COR). Shortly, cells were lysed, and nuclear extract was gained as described above. 5 *μ*g nuclear extract (14 *μ*l including water and sample) was incubated with 2 *μ*l 10x binding buffer (100 mM Tris, 500 mM KCl, and 10 mM DTT; pH 7.5), 1 *μ*l poly(dI·dC) (1 *μ*g/*μ*l in 10 mM Tris and 1 mM EDTA; pH 7.5), 2 *μ*l 25 mM DTT/2.5% Tween® 20 (all components of the Odyssey® EMSA Buffer Kit #829-07910), and 1 *μ*l IRDye® NF*κ*B Oligonucleotide for 30 min in the dark. After that, 10x Orange loading buffer was added, and the total mixture was loaded onto a 4% native polyacrylamide gel. Detection was performed with an Odyssey® Infrared Imaging System at 700 nm.

### 2.7. ROS Measurements with Chemiluminescence

After polarization, macrophages were dissociated from the plate with Ca^2+^-free EDTA/EGTA (Versene). ROS levels were assessed in intact cells with either L-012 (200 *μ*mol/l) or luminol (100 *μ*mol/l)/horseradish peroxidase (HRP at 1 U/ml) in a Berthold 6-channel luminometer (LB9505, Berthold, Wildbad, Germany). All measurements were performed in the HEPES-Tyrode (HT) buffer containing (in mmol/l) 137 NaCl, 2.7 KCl, 0.5 MgCl_2_, 1.8 CaCl_2_, 5 glucose, 0.36 NaH_2_PO_4_, and 10 HEPES. Substances added during the experiment were used as follows: PMA 100 nM, DPI 10 *μ*M, L-NAME 300 *μ*M, PEG-catalase 250 U/ml, and PEG-SOD 50 U/ml.

### 2.8. Flow Cytometry

Tumor tissue was lysed with the aid of the tumor dissociation kit, mouse (Miltenyi) according to the manufacturer protocol. 3^∗^10*E*6 cells were used for flow cytometry. Cells were pelleted by centrifugation at 500 g and resuspended in 100 *μ*l PBS+0.5% BSA. CD16/32 blocking antibody was added to the cells for 15 minutes subsequently followed by a 15-minute incubation with the prepared mastermix of all antibodies indicated in Table 2. After staining, FACS flow was added; cells were centrifuged and resuspended in FACS flow for measurement. Samples were acquired with a LSRII/Fortessa flow cytometer (BD Biosciences) and analyzed using FlowJo software Vx (Treestar).

### 2.9. Statistics

All values are displayed as mean ± SEM. Statistical analysis was performed by ANOVA followed by LSD post hoc testing or by the *t* test if appropriate. Densitometry was performed with the aid of the Odyssey software. A *p* value of less than 0.05 was considered statistically significant.

## 3. Results

### 3.1. Nox4 Deficiency Promotes Inflammation in Murine Fibrosarcomas

**Figure 1:**
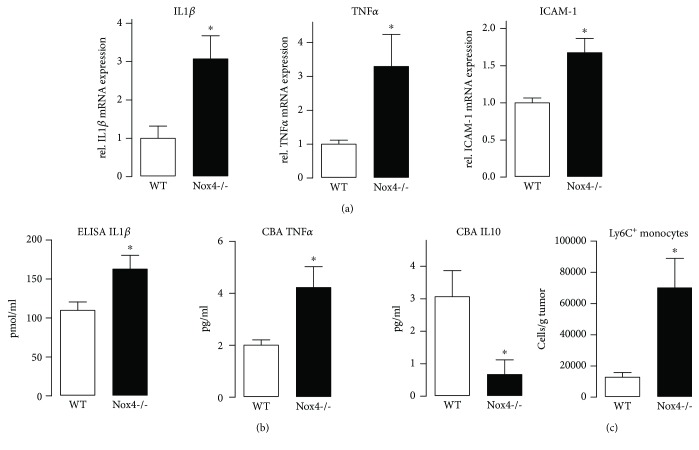
Nox4 deficiency favors inflammation in a murine tumor model. (a) Proinflammatory markers including the cytokines IL1*β* and TNF*α* and the adhesion molecule ICAM-1 were quantified by RT-qPCR in tumor tissue of WT and Nox4-/- mice. (b) Proinflammatory markers IL1*β* (ELISA) and TNF*α* as well as IL10 (CBA: cytometric bead assay) as anti-inflammatory markers were measured in tumor tissue, *n* = 3; ^∗^ *p* < 0.05. (c) Single-cell suspension of tumor tissue was analyzed by FACS for Ly6C^+^ monocytes; ^∗^ *p* < 0.05, *n* = 5-10.

In a murine fibrosarcoma model, the absence of Nox4 forces tumor growth [16]. Simultaneously, mRNA abundance of proinflammatory cytokines such as IL1*β* and TNF*α* and other markers of inflammation, such as ICAM-1, was elevated (Figure 1(a)). Accordingly, IL1*β* and TNF*α* were elevated, when measured with an ELISA or a cytometric bead assay, respectively. In contrast, the anti-inflammatory cytokine IL10 was strongly reduced in tumors of Nox4-deficient mice (Figure 1(b)). These data point towards a more severe inflammation in tumors of Nox4-/- mice. However, the total number of immune cells per tissue was similar in wild type and Nox4-/- mice (Supplemental Figure 1) as measured by flow cytometry. Therefore, we analyzed the number of proinflammatory macrophages, identified by high expression of the surface marker Ly6C [17], and found a substantial increase in Ly6C^hi^ monocytes in the tumors of Nox4-/- mice (Figure 1(c)). When we further analyzed the tumor tissue for pro- and anti-inflammatory markers, we observed a trend towards more inflammation, together with lower expression of markers typical for M(IL4+IL13)-polarized macrophages (Supplemental Figure 2). Accordingly, we conclude that the absence of Nox4 favors the polarization of macrophages towards a proinflammatory phenotype, which was further investigated.

### 3.2. Loss of Nox4 Promotes M(LPS+IFN*γ*) Polarization of Macrophages

**Figure 2:**
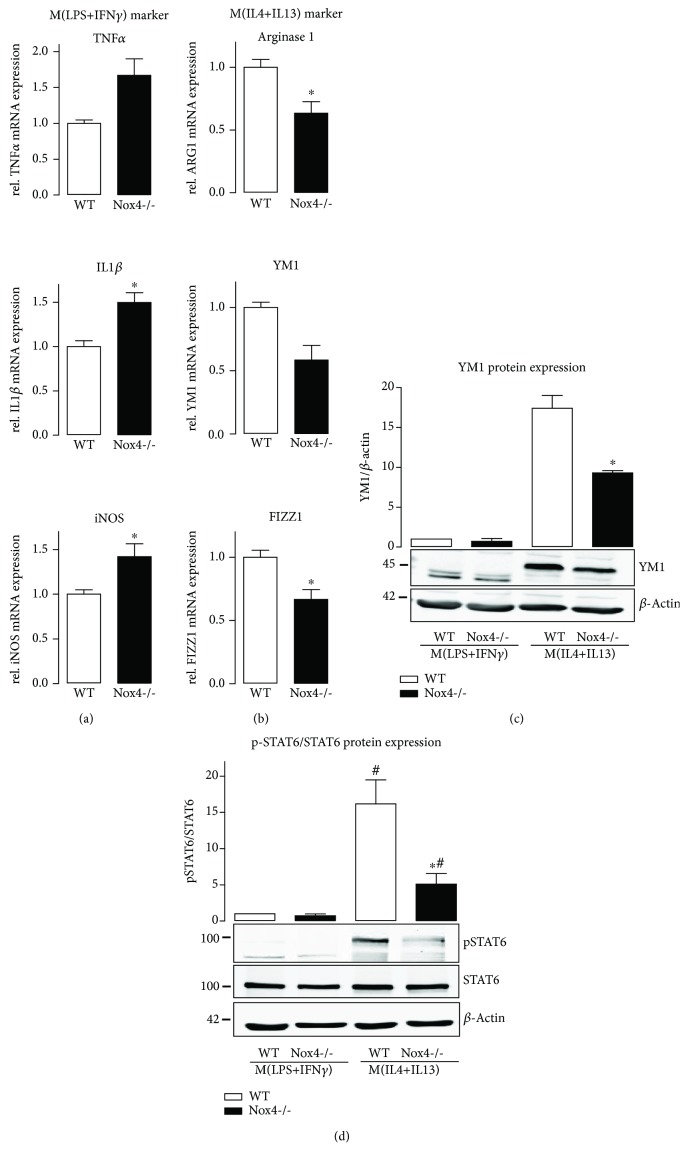
Nox4 knockout leads to a decreased M(IL4+IL13) polarization of macrophages. The specific M(LPS+IFN*γ*) markers IL1*β*, TNF*α*, and iNOS (a) and specific M(IL4+IL13) markers arginase 1, YM1, and FIZZ1 (b) were quantified by RT-qPCR after stimulation with cytokines polarizing the bone marrow-derived macrophages from WT and Nox4-/- mice to M(LPS+IFN*γ*) or M(IL4+IL13) phenotype. Protein expression of the M(IL4+IL13) marker YM1 (c) and the ratio of pSTAT6 to STAT6 (d) as determined by Western Blot; ^∗^ *p* < 0.05 WT vs. Nox4-/- and #*p* < 0.05 WT/Nox4-/- M(LPS+IFN*γ*) vs. WT/Nox4-/- M(IL4+IL13), *n* = 5-6.

Human and murine macrophages were generated and analyzed for the expression of individual NADPH oxidases. As expected, Nox2 expression was the highest in both macrophage populations, followed by Nox4 and Nox1 (Supplemental Figure 3). In order to analyze if Nox4 influences macrophage polarization, we isolated monocytes from bone marrow of wild type and Nox4-deficient mice, challenged them (with M-CSF) to become macrophages, and eventually polarized them to either M(LPS+IFN*γ*) or M(IL4+IL13) phenotype. Nox4 knockout promoted the expression of M(LPS+IFN*γ*) macrophage markers including TNF*α* and IL1*β* (Figure 2(a)), whereas typical M(IL4+IL13) markers were significantly downregulated (Figure 2(b)). This effect was mediated by H_2_O_2_, the product of Nox4: external H_2_O_2_ or increased intracellular H_2_O_2_ formation via PMA-induced activation of Nox2 and conversion of the resulting ·O_2_^−^ into H_2_O_2_ by SOD induced M(IL4+IL13) polarization. Depletion of H_2_O_2_ by catalase forces the expression of M(LPS+IFN*γ*) markers, both without any further treatment with cytokines (Supplemental Figure 4). Exemplary verification of the PCR results on the protein level revealed the same effect for the M(IL4+IL13) marker YM1 (Figure 2(c)). STAT6 is one of the main transcription factors involved in the expression of M(IL4+IL13) markers. In line with the decreased level of M(IL4+IL13) markers in Nox4-/- cells, phosphorylation of STAT6 was attenuated (Figure 2(d)). In order to analyze whether or not the effects seen are specific for Nox4, macrophage polarization was determined in Nox2- and Nox1-deficient macrophages as well. In contrast to Nox4-/- macrophages, loss of Nox2 induced a small but significant reduction in M(LPS+IFN*γ*) polarization with no effect on M(IL4+IL13) polarization or STAT6 phosphorylation (Supplemental Figure 5). Knockout of Nox1 had no effect on macrophage polarization, compared to wild type littermates (Supplemental Figure 6).

### 3.3. Formation of Reactive Oxygen Species upon M(LPS+IFN*γ*) Polarization Is Elevated in the Absence of Nox4

**Figure 3:**
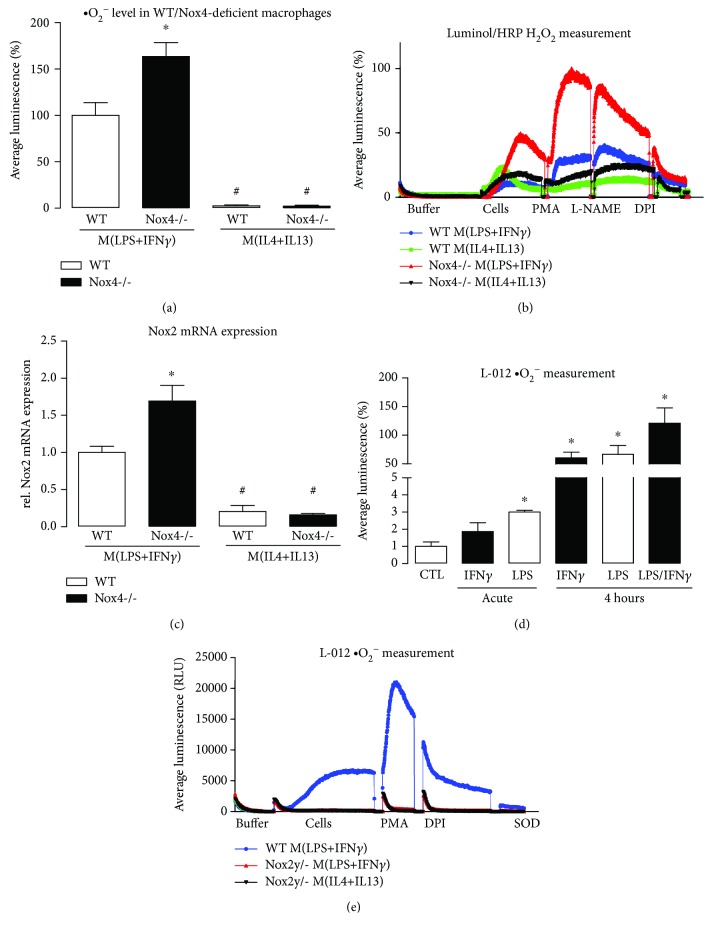
ROS measurements reveal increased ROS production in Nox4-deficient cells due to an increase in Nox2. Superoxide anion production measured with L-012 (a) and hydrogen peroxide levels measured with luminol and HRP (b) in polarized macrophages of wild type and Nox4 knockout mice. (c) RT-qPCR for Nox2 mRNA expression in polarized macrophages of WT and Nox4-deficient animals; ^∗^ *p* < 0.05 (*n* = 3 − 8). (d) Superoxide anion production, as measured with L-012 in WT macrophages with or without LPS (10 *μ*g/ml) and IFN*γ* (100 U/ml) directly after stimulation or 4 hours after addition. (e) Superoxide anion production in polarized WT and Nox2-deficient macrophages; ^∗^ *p* < 0.05 WT vs. Nox4-/- or treated vs. CTL and #*p* < 0.05 WT/Nox4-/- M(LPS+IFN*γ*) vs. WT/Nox4-/- M(IL4+IL13) (*n* = 3-5).

Several publications indicate that polarization of macrophages is dependent on ROS production and simultaneously forces ROS formation [18]. Polarization of macrophages towards the proinflammatory M(LPS+IFN*γ*) phenotype resulted in an increase in superoxide anion as well as in hydrogen peroxide production compared to M(IL4+IL13)-polarized macrophages (Figures 3(a) and 3(b)). Surprisingly, the absence of Nox4 further increased ROS formation in M(LPS+IFN*γ*)-polarized macrophages (Figures 3(a) and 3(b)). A major source of ROS in M(LPS+IFN*γ*)-polarized macrophages is Nox2, whose expression was elevated in Nox4-deficient M(LPS+IFN*γ*)-polarized macrophages (Figure 3(c)). Accordingly, when measuring ·O_2_^−^ in a more specific way with the aid of L-012 in intact cells, we found that both LPS and IFN*γ* separately increase the level of ·O_2_^−^ production in macrophages as well as the combination of both (Figure 3(d)). Knockout of Nox2 in macrophages completely abolished L-012 detectable ·O_2_^−^ formation (Figure 3(e)). In conclusion, the increase in Nox2 expression, which predominantly produces ·O_2_^−^ over H_2_O_2_, indicates that Nox2 is the major source of ROS in M(LPS+IFN*γ*)-polarized macrophages.

### 3.4. Nox4 Mediates the Proinflammatory Macrophage Polarization via Activation of NF*κ*B

Inflammation is often associated with an increased activity of NF*κ*B [19]. Indeed, TNF*α* and IL1*β* as well as ICAM-1 and Nox2 are target genes of NF*κ*B. Therefore, we analyzed the potential role of Nox4 in NF*κ*B activation in the course of macrophage polarization.

**Figure 4:**
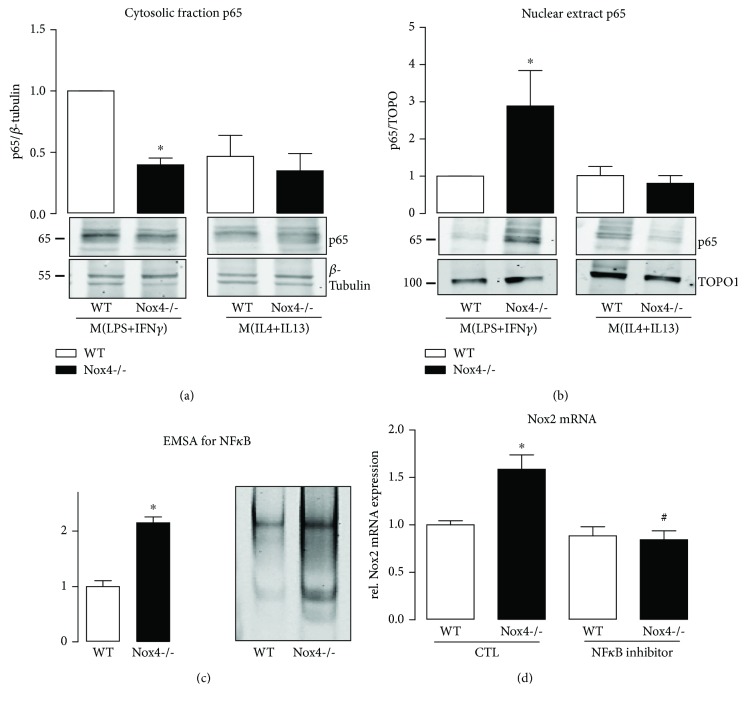
Increased NF*κ*B activation in M(LPS+IFN*γ*)-polarized macrophages of Nox4-/- is responsible for elevated Nox2 expression. Translocation of p65 was analyzed by Western Blot in the cytosol (a) and nuclear fraction (b) of M(LPS+IFN*γ*)- and M(IL4+IL13)-polarized macrophages of WT and Nox4-/- mice. (c) Electrophoretic mobility shift assay for NF*κ*B was performed in M(LPS+IFN*γ*)-polarized macrophages of WT and Nox4-/- animals. The left bar graph shows quantification, and the right bar graph representative shift. (d) Nox2 mRNA expression was quantified by RT-qPCR after M(LPS+IFN*γ*) polarization with and without an NF*κ*B inhibitor (30 ng/ml, 1 h pretreatment before polarization); ^∗^ *p* < 0.05 WT vs. Nox4-/- and #*p* < 0.05 CTL vs. NF*κ*B inhibitor (*n* = 3-8). TOPO: topoisomerase I.

M(LPS+IFN*γ*) polarization was accompanied by an increased translocation of p65 from the cytosol to the nucleus in the Nox4-deficient macrophages when compared to wild type cells (Figures 4(a) and 4(b)). However, nuclear translocation alone is not sufficient as the indicator of a transcription factor activity. In order to test for both, NF*κ*B nuclear translocation and DNA binding activity, an electro mobility shift assay (EMSA) was utilized. Activity of NF*κ*B was enhanced in M(LPS+IFN*γ*) macrophages in the absence of Nox4 (Figure 4(c)). In M(IL4+IL13)-polarized macrophages, no such effect of Nox4 was observed.

### 3.5. Activated NF*κ*B Promotes Nox2 Expression in the Absence of Nox4

NF*κ*B is one of the transcription factors that control Nox2 expression. We therefore hypothesized that elevated activation of NF*κ*B in the absence of Nox4 promotes Nox2 expression during macrophage M(LPS+IFN*γ*) polarization. The upregulation of Nox2 however is not accompanied by an elevated expression of its cytosolic subunits or antioxidative enzymes such as SOD1 or 3 in wild type vs. Nox4-/- cells (Supplemental Figure 7). Treatment of the cells with an NF*κ*B inhibitor prevented the increase in p65 nuclear translocation (Supplemental Figure 8), and Nox2 expression was reduced in Nox4-/- macrophages to the level similar to that of the wild type, when cells were pretreated with the NF*κ*B inhibitor prior to M(LPS+IFN*γ*) polarization (Figure 4(d)). NF*κ*B acts in concert with other transcription factors to regulate the expression of Nox2 [20]. One of which is the redox-sensitive zinc-finger transcription factor Yin Yang 1 (YY1), which directly controls the activity of NF*κ*B [21]. As such, YY1 represents a potential target of Nox4-derived ROS, which is upstream of NF*κ*B and controls Nox2 expression. A significant increase in the YY1 protein level was observed in M(LPS+IFN*γ*)-polarized Nox4-/- macrophages; which was not the case for M(IL4+IL13)-polarized macrophages (Supplemental Figure 9A). Inhibition of Nox4 with GKT137928 in Nox2-deficient macrophages results in a small but significant increase in LPS and IFN*γ*-induced M(LPS+IFN*γ*) polarization. Under basal conditions, treatment with GKT only increased iNOS, compared to DMSO-treated samples. Those results indicate that inhibition of Nox4 favors M(LPS+IFN*γ*) polarization even in the absence of Nox2 (Supplemental Figure 10). The interpretation of this result could be that NF*κ*B even in the absence of Nox2 promotes M(LPS+IFN*γ*) polarization in macrophages. Although many studies provide evidence for the involvement of NF*κ*B in macrophage polarization, the exact role of NF*κ*B and its effects besides induction of Nox2 are unclear. Therefore, investigation of how NF*κ*B triggers M(LPS+IFN*γ*) polarization in the absence of Nox2 would be worth a second study. Another transcription factor involved in M(LPS+IFN*γ*) polarization is STAT1 [22]. We therefore checked for a potential effect of Nox4 on phosphorylation of STAT1 in M(LPS+IFN*γ*) polarization without observing any effect of Nox4 (Supplemental Figure 9B). Thus, Nox4 appears to selectively regulate the activity of NF*κ*B and potentially YY1. In conclusion, the absence of Nox4 promotes Nox2 expression and subsequently M(LPS+IFN*γ*) polarization of macrophages.

### 3.6. Pharmacological Inhibition of Nox4 Promotes M(LPS+IFN*γ*) Polarization of Human Macrophages

**Figure 5:**
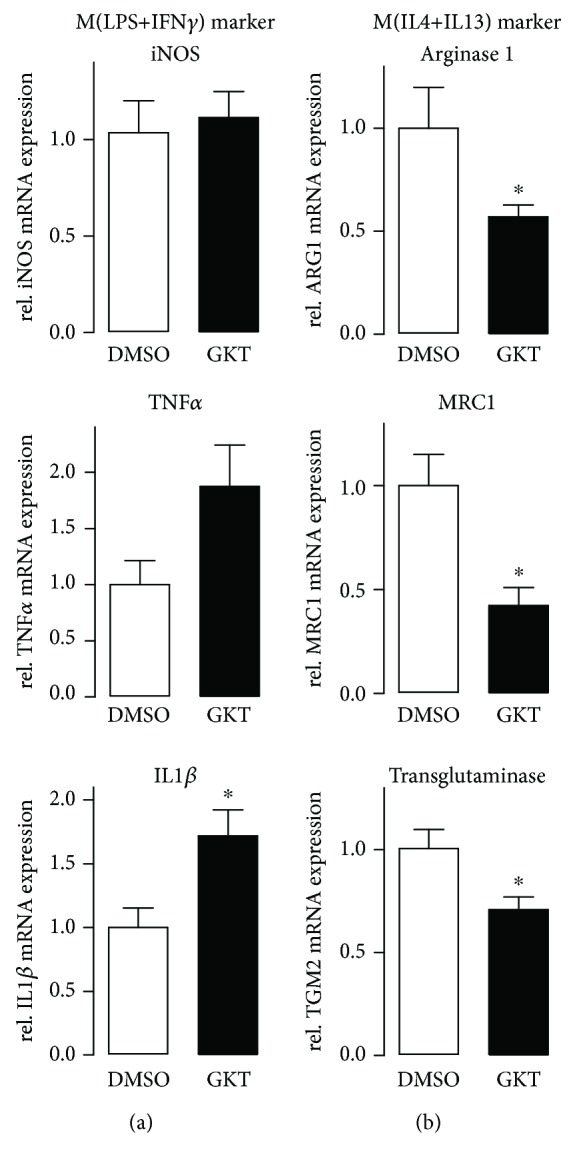
Treatment of human macrophages with the Nox4 inhibitor GKT137928 forces M(LPS+IFN*γ*) polarization. The specific M(LPS+IFN*γ*) markers iNOS, TNF*α*, and IL1*β* (a) and M(IL4+IL13) markers arginase 1, MRC1, and transglutaminase 2 (b) were quantified by RT-qPCR. Cells were preincubated with Nox4 inhibitor GKT (10 *μ*M, 2 h) followed by stimulation with cytokines polarizing human macrophages; ^∗^ *p* < 0.05 (*n* = 6).

In order to determine whether our findings in a mouse can be translated to human cells, human macrophages generated from peripheral blood of healthy donors were analyzed. Inhibition of Nox4 was achieved by treatment of the cells with the Nox1/Nox4 inhibitor GKT137928. Upon treatment of the macrophages with GKT137928, an increased M(LPS+IFN*γ*) polarization was observed. This was accompanied by a decrease in M(IL4+IL13) polarization (Figure 5). As shown above in the murine system, knockout of Nox1 has no influence on macrophage polarization. Therefore, we assume that usage of the inhibitor will mainly affect Nox4-mediated signaling in the process of polarization. We conclude that the findings in the murine system also apply to the human system.

## 4. Discussion

Macrophages are a heterogeneous population of cells. Generally, they can be categorized into two discrete subsets as either classically activated M1 or alternatively activated M2 macrophages, herein referred to as M(LPS+IFN*γ*) or M(IL4+IL13). In this context, M(LPS+IFN*γ*) macrophages represent proinflammatory “killers,” while M(IL4+IL13) macrophages serve as “builders” in inflammatory wound repair. This polarization of the macrophage population results from interactions with other cells or molecules within the host tissues [23]. In previous work, we found that knockout of the NADPH oxidase Nox4 enhances inflammation and tumorigenesis [16, 24]. In an angiotensin II-induced model of vascular dysfunction, loss of Nox4 promoted the expression of the proinflammatory cytokines IL6 and IL1*β* [12]. The present study underlines the protective potential of Nox4 in inflammation, as it promotes M(IL4+IL13) polarization of macrophages. Our results were confirmed in a very recent study in a myocardial infarction model, where overexpression of Nox4 promoted M(IL4+IL13) polarization of cardiac macrophages and protects from postinfarction remodeling [25].

The balance between activation of STAT1 and STAT3/STAT6 plays a crucial role in macrophage polarization: a predominance of STAT1 activation promotes M(LPS+IFN*γ*), while STAT3/STAT6 activation increases M(IL4+IL13) macrophage polarization [26]. In fact, STAT6 is the most important transcription factor regulating M(IL4+IL13) polarization of macrophages [27], and phosphorylation of STAT6 can be regulated by redox-sensitive phosphatases [28]. Therefore, it is likely that Nox4-derived H_2_O_2_ at least contributes to STAT6 phosphorylation and thereby to M(IL4+IL13) polarization. Importantly, STAT6 suppresses NF*κ*B activation via Klf4. Here, we provide evidence that Nox4 deficiency prevents STAT6 phosphorylation and supports NF*κ*B activation. NF*κ*B has been shown to promote M(LPS+IFN*γ*) polarization of phagocytes [29]. Besides regulation by STAT6/Klf4, the activity of NF*κ*B is redox sensitive and potentially regulated by NADPH oxidases [30]. Both, increased NF*κ*B and reduced phosphorylation of STAT6, inhibit the polarization of macrophages towards the M(IL4+IL13) phenotype. Consequently, since there exists an intrinsic balance, less force in the direction of M(IL4+IL13) will lead to more M(LPS+IFN*γ*) polarization, as observed in the current study. Since Nox4 produces H_2_O_2_, a Nox4 knockout would naturally lead to a reduced production of H_2_O_2_. Therefore, our data can be supported by a finding concerning CuZn-SOD, an enzyme catalyzing the conversion of superoxide anion to hydrogen peroxide. Consequently, less H_2_O_2_ is formed in the absence of CuZn-SOD. Knockout of this enzyme promotes M(IL4+IL13) polarization of macrophages [8], favoring the hypothesis that indeed H_2_O_2_ mediates the effect of Nox4.

Different to Nox4, Nox2 produces superoxide anions (·O_2_^−^), and knockout of Nox2 results in a decreased M(LPS+IFN*γ*) polarization. In contrast, Nox2, via production of superoxide anions, contributes to M(LPS+IFN*γ*) polarization [6]. We observed not only a reduced M(LPS+IFN*γ*) polarization in Nox2-deficient macrophages but also an increase in Nox2 expression and subsequently ·O_2_^−^ formation, in Nox4 knockout macrophages. This is potentially a consequence of the abovementioned enhanced NF*κ*B activation in the absence of Nox4, as Nox2 expression is enhanced by NF*κ*B [31].

We conclude that the specific types of ROS, such as H_2_O_2_ or ·O_2_^−^, differentially contribute to M(LPS+IFN*γ*) or M(IL4+IL13) macrophage polarization. Importantly, knockout of Nox4 not only favors M(LPS+IFN*γ*) polarization but also results in an increased expression of Nox2 in M(LPS+IFN*γ*)-polarized macrophages.

## Figures and Tables

**Table 1:** Primer sequences used.

Gene	Forward (5′ to 3′)	Reverse (5′ to 3′)
m TNF*α*	CCCGACTACGTGCTCCTCACC	CTCCAGCTGGAAGACTCCTCCCAG
m IL1*β*	GACCTTCCAGGATGAGGACATGAG	GGTGGGTGTGCCGTCTTTCATTAC
m ICAM-1	TGGCCTGGGGGATGCACACT	GGCTGTAGGTGGGTCCGGGT
m iNOS	TGAAGAAAACCCCTTGTGCT	TTCTGTGCTGTCCCAGTGAG
m YM1	CTGGAATTGGTGCCCCTACAA	TCATAACCAACCCACTCATTACC
m FIZZ1	GCAACTGCCTGTGCTTACTC	AGAAGCAGGGTAAATGGGCAA
m ARG1	GACAGGGCTCCTTTCAGGAC	CTTGGGAGGAGAAGGCGTTT
m Nox2	GTGCACCATGATGAGGAGAA	TTGCAATGGTCTTGAACTCG
m Nox1	CGCTCCCAGCAGAAGGTCGTGATTACCAAGG	GGAGTGACCCCAATCCCTGCCCCAACCA
m Nox4	TGTTGGGCCTAGGATTGTGTT	AGGGACCTTCTGTGATCCTCG
h Nox2	GTCACACCCTTCGCATCCATTCTCAAGTCAGT	CTGAGACTCATCCCAGCCAGTGAGGTAG
h Nox1	TTCACCAATTCCCAGGATTGAAGTGGATGGTC	GACCTGTCACGATGTCAGTGGCCTTGTCAA
h Nox4	CTGGAGGAGCTGGCTCGCCAACGAAG	GTGATCATGAGGAATAGCACCACCACCATGCAG
h iNOS	GACCTGGGACCCGCACCACT	AGGATGGTGGCACGGCTGGA
h TNF*α*	TGGAGAAGGGTGACCGACTC	TCCTCACAGGGCAATGATCC
h IL1*β*	CTGTACGATCACTGAACTGC	CACCACTTGTTGCTCCATATC
h ARG1	TTCTCAAAGGGACAGCCACG	AGCACCAGGCTGATTCTTCC
h MRC1	GGAGTGATGGAACCCCAGTG	CTGTCCGCCCAGTATCCATC
h TGM2	TTCAGGGTACAAACTGAGGCTGCT	TATTCAAGTTCACCCACTGGCCCT

**Table 2:** Antibodies used.

Antigen	Dye
CD3	PE-CF594
CD4	V500
CD8	BV650
CD11b	eFluor 605
CD11c	Alexa Fluor 700
CD19	APC-H7
CD45	VioBLue
CD49b	PE-CF594
F4/80	PE-Cy7
HLA-DR (MHCII)	APC-H7
Ly-6C	PerCP-Cy5.5
Ly-6G	APC-Cy7
Siglec H	FITC

## Data Availability

All data used to support the findings of this study are included within the article or the supplements.

## Supplementary Materials

Supplementary Materials Supplemental Figure 1: FACS analysis of tumor tissue revealed only a tendency for differences in B cells within Nox4-deficient tumors. (A) Fibrosarcoma tissues of wild type and Nox4-/- mice were analyzed for cell composition with FACS using specific antibodies for cells indicated. The table contains the different T cell populations in cells/g tumor tissue, no statistical differences (*n* = 6-10). Supplemental Figure 2: tumor tissue was analyzed for different inflammatory and anti-inflammatory markers. (A) Fibrosarcoma tissues of wildtype and Nox4-/- mice were analyzed for proinflammatory markers iNOS and CD163, anti-inflammatory markers FIZZ1, arginase 1, and YM1, and tissue remodeling markers MMP9 and collagens I and III with RT-qPCR; ^∗^ *p* < 0.05 (*n* = 6-10). Supplemental Figure 3: NADPH oxidase expression in isolated murine and human macrophages. Nox1, Nox2, and Nox4 expressions were determined by RT-qPCR in isolated murine (A) and human (B) macrophages, and corresponding CT values were included (*n* = 3). Supplemental Figure 4: H_2_O_2_ mediates polarization of macrophages without cytokine stimulation. (A) WT macrophages were treated with basal medium or IL4 and IL13 to polarize. For polarization without cytokines, cells were treated with 5 *μ*M H_2_O_2_ or PEG-SOD (50 U/ml) and PMA (100 nM) for 4 h, and polarization markers ARG1, FIZZ1, and YM1 were quantified with RT-qPCR; ^∗^ *p* < 0.05 (*n* = 6). (B) WT macrophages were treated with basal medium or LPS and IFN*γ* or PEG-catalase (500 U/ml) for 4 h to polarize, followed by subsequent analysis of polarization markers TNF*α*, IL1*β*, and iNOS; ^∗^ *p* < 0.05 (*n* = 3). Supplemental Figure 5: Nox2 knockout decreases M(LPS+IFN*γ*) polarization of macrophages. The specific M(LPS+IFN*γ*) markers IL1*β*, TNF*α*, and iNOS (A) and specific M(IL4+IL13) markers arginase 1, YM1, and FIZZ1 (B) were quantified by RT-qPCR after stimulation with cytokines polarizing the bone marrow-derived macrophages from Nox2KO/C57Bl6J mice to M(LPS+IFN*γ*) or M2(IL4+IL13) phenotype. Protein expression of the M(IL4+IL13) marker YM1 (C) and the ratio of pSTAT6 to STAT6 (D) as determined by Western Blot; ^∗^ *p* < 0.05 and #*p* < 0.05 WT/Nox2y/- M(LPS+IFN*γ*) vs. WT/Nox2y/- M(IL4+IL13) (*n* = 4 − 8). Supplemental Figure 6: deficiency in Nox1 does not affect polarization of macrophages. The specific M(LPS+IFN*γ*) markers IL1*β*, TNF*α*, and iNOS (A) and specific M(IL4+IL13) markers arginase 1, YM1, and FIZZ1 (B) were quantified by RT-qPCR after stimulation with cytokines polarizing the bone marrow-derived macrophages from Nox1KO/C57Bl6J mice to M(LPS+IFN*γ*) or M2(IL4+IL13) phenotype. Protein expression of the M(IL4+IL13) marker YM1 (C) and the ratio of pSTAT6 to STAT6 (D) as determined by Western Blot; ^∗^ *p* < 0.05 and #*p* < 0.05 WT/Nox1y/- M(LPS+IFN*γ*) vs. WT/Nox1y/- M(IL4+IL13) (*n* = 3 − 6). Supplemental Figure 7: SOD and Nox2 cytosolic subunit expressions in WT and Nox4-deficient macrophages. SOD1 (A) and SOD3 (B) expressions were analyzed in WT and Nox4-/- M(LPS+IFN*γ*)- and M(IL4+IL13)-polarized macrophages by RT-qPCR. Expressions of cytosolic Nox2 subunits (C: p40phox, D: p47phox, and E: p67phox) and Nox1 (F) were analyzed in WT and Nox4-/- M(LPS+IFN*γ*)- and M(IL4+IL13)-polarized macrophages using RT-qPCR; ^∗^ *p* < 0.05 WT/Nox4-/- M(LPS+IFN*γ*) vs. WT/Nox4-/- M(IL4+IL13) (*n* = 5-8). Supplemental Figure 8: NF*κ*B inhibition prevents p65 translocation into the nucleus in M(LPS+IFN*γ*)-polarized macrophages of Nox4-/-. P65 expression in cytosol (A) and nucleus (B) was assessed with Western Blot after M(LPS+IFN*γ*) polarization with and without treatment of NF*κ*B inhibitor (30 ng/ml, 1 h pretreament before M(LPS+IFN*γ*) polarization); ^∗^ *p* < 0.05 WT vs. Nox4-/- and #*p* < 0.05 CTL vs. NF*κ*B inhibitor (*n* = 3-8). TOPO: topoisomerase I. Supplemental Figure 9: YY1 is increased in Nox4-deficient macrophages after M(LPS+IFN*γ*) polarization. (A) YY1 expression was determined by Western Blot after polarization of WT and Nox4-/- macrophages. (B) Phosphorylation of pSTAT1 and total STAT1 quantified by Western Blot in M(LPS+IFN*γ*)- and M(IL4+IL13)-polarized macrophages of WT and Nox4-deficient animals; ^∗^ *p* < 0.05 WT vs. Nox4-/- and #*p* < 0.05 WT/Nox4-/- M(LPS+IFN*γ*) vs. WT/Nox4-/- M(IL4+IL13) (*n* = 3-5). Supplemental Figure 10: inhibition of Nox4 in Nox2-deficient macrophages elevates M(LPS+IFN*γ*) polarization in M(LPS+IFN*γ*)-polarized macrophages. Nox2-deficient macrophages were treated with Nox4 inhibitor GKT (10 *μ*M) 2 h prior to cell polarization to M(LPS+IFN*γ*) or only control medium (CTL). M(LPS+IFN*γ*) markers TNF*γ*, IL1*β*, and iNOS were evaluated using RT-qPCR; ^∗^ *p* < 0.05 DMSO vs. GKT and #*p* < 0.05 DMSO/GKT CTL vs. DMSO/GKT M(LPS+IFN*γ*) (*n* = 3).
