# Supplementary material for: The NADPH Oxidase Nox4 Controls Macrophage Polarization in an NFκB-Dependent Manner
Source: Oxid Med Cell Longev. 2019 Apr 18;2019:3264858. doi: 10.1155/2019/3264858 (PMC6501210; doi:10.1155/2019/3264858)
Supplement: Supplementary Materials — Supplemental Figure 1: FACS analysis of tumor tissue revealed only a tendency for differences in B cells within Nox4-deficient tumors. (A) Fibrosarcoma tissues of wild type and Nox4-/- mice were analyzed for cell composition with FACS using specific antibodies for cells indicated. The table contains the different T cell populations in cells/g tumor tissue, no statistical differences (n = 6-10). Supplemental Figure 2: tumor tissue was analyzed for different inflammatory and anti-inflammatory markers. (A) Fibrosarcoma tissues of wildtype and Nox4-/- mice were analyzed for proinflammatory markers iNOS and CD163, anti-inflammatory markers FIZZ1, arginase 1, and YM1, and tissue remodeling markers MMP9 and collagens I and III with RT-qPCR; ∗ p < 0.05 (n = 6-10). Supplemental Figure 3: NADPH oxidase expression in isolated murine and human macrophages. Nox1, Nox2, and Nox4 expressions were determined by RT-qPCR in isolated murine (A) and human (B) macrophages, and corresponding CT values were included (n = 3). Supplemental Figure 4: H2O2 mediates polarization of macrophages without cytokine stimulation. (A) WT macrophages were treated with basal medium or IL4 and IL13 to polarize. For polarization without cytokines, cells were treated with 5 μM H2O2 or PEG-SOD (50 U/ml) and PMA (100 nM) for 4 h, and polarization markers ARG1, FIZZ1, and YM1 were quantified with RT-qPCR; ∗ p < 0.05 (n = 6). (B) WT macrophages were treated with basal medium or LPS and IFNγ or PEG-catalase (500 U/ml) for 4 h to polarize, followed by subsequent analysis of polarization markers TNFα, IL1β, and iNOS; ∗ p < 0.05 (n = 3). Supplemental Figure 5: Nox2 knockout decreases M(LPS+IFNγ) polarization of macrophages. The specific M(LPS+IFNγ) markers IL1β, TNFα, and iNOS (A) and specific M(IL4+IL13) markers arginase 1, YM1, and FIZZ1 (B) were quantified by RT-qPCR after stimulation with cytokines polarizing the bone marrow-derived macrophages from Nox2KO/C57Bl6J mice to M(LPS+IFNγ) or M2(IL4+IL13) phenotype. [file 3264858.f1.pptx]

## Slide 1
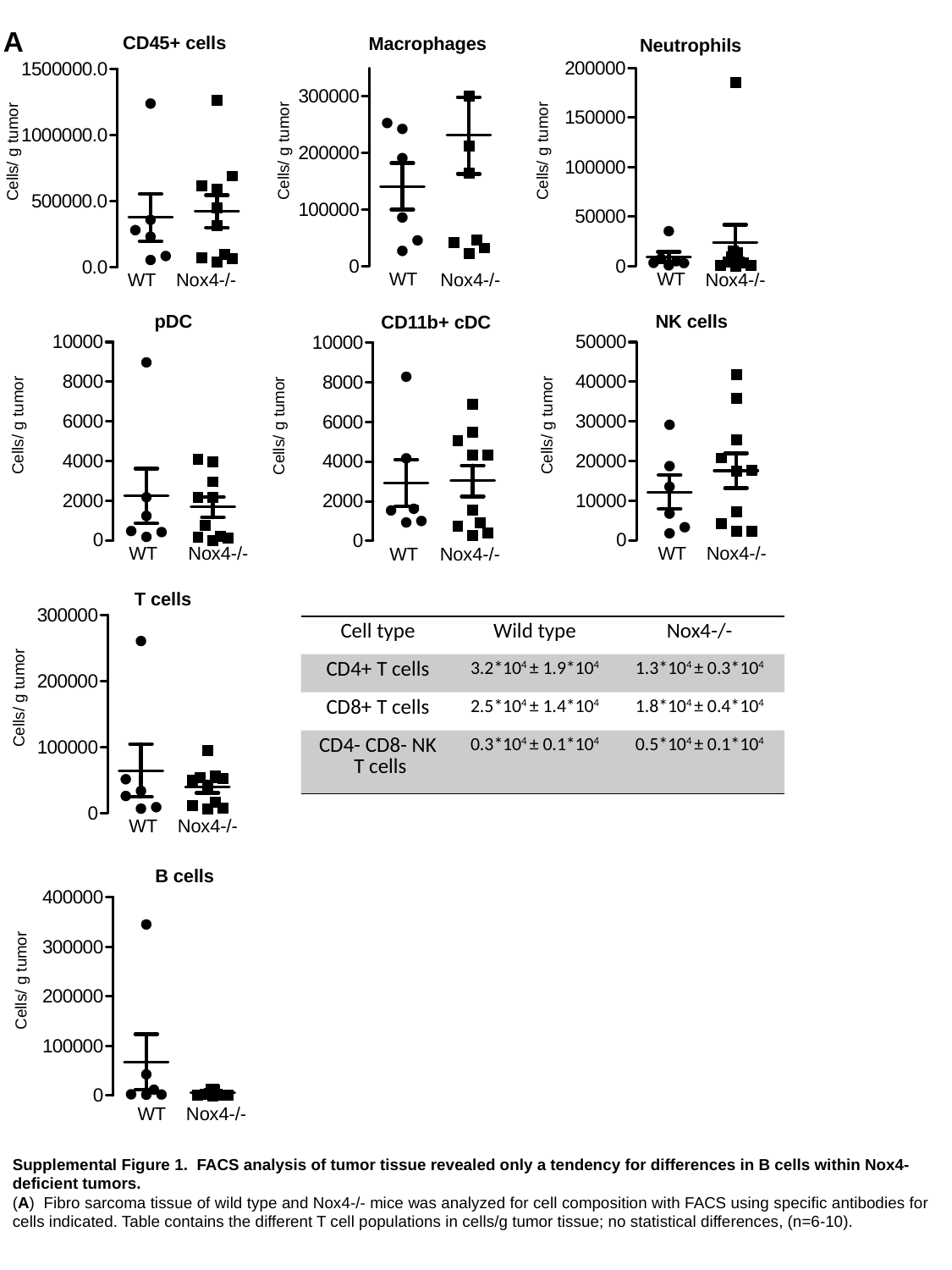

A
CD45+ cells
Macrophages
Neutrophils
Cells/ g tumor
Cells/ g tumor
Cells/ g tumor
WT
WT
Nox4-/-
Nox4-/-
WT
Nox4-/-
pDC
NK cells
CD11b+ cDC
Cells/ g tumor
Cells/ g tumor
Cells/ g tumor
WT
WT
Nox4-/-
Nox4-/-
WT
Nox4-/-
T cells
| Cell type | Wild type | Nox4-/- |
| --- | --- | --- |
| CD4+ T cells | 3.2\*104 ± 1.9\*104 | 1.3\*104 ± 0.3\*104 |
| CD8+ T cells | 2.5\*104 ± 1.4\*104 | 1.8\*104 ± 0.4\*104 |
| CD4- CD8- NK T cells | 0.3\*104 ± 0.1\*104 | 0.5\*104 ± 0.1\*104 |
Cells/ g tumor
WT
Nox4-/-
B cells
Cells/ g tumor
WT
Nox4-/-
Supplemental Figure 1. FACS analysis of tumor tissue revealed only a tendency for differences in B cells within Nox4-deficient tumors.
(A) Fibro sarcoma tissue of wild type and Nox4-/- mice was analyzed for cell composition with FACS using specific antibodies for cells indicated. Table contains the different T cell populations in cells/g tumor tissue; no statistical differences, (n=6-10).

## Slide 2
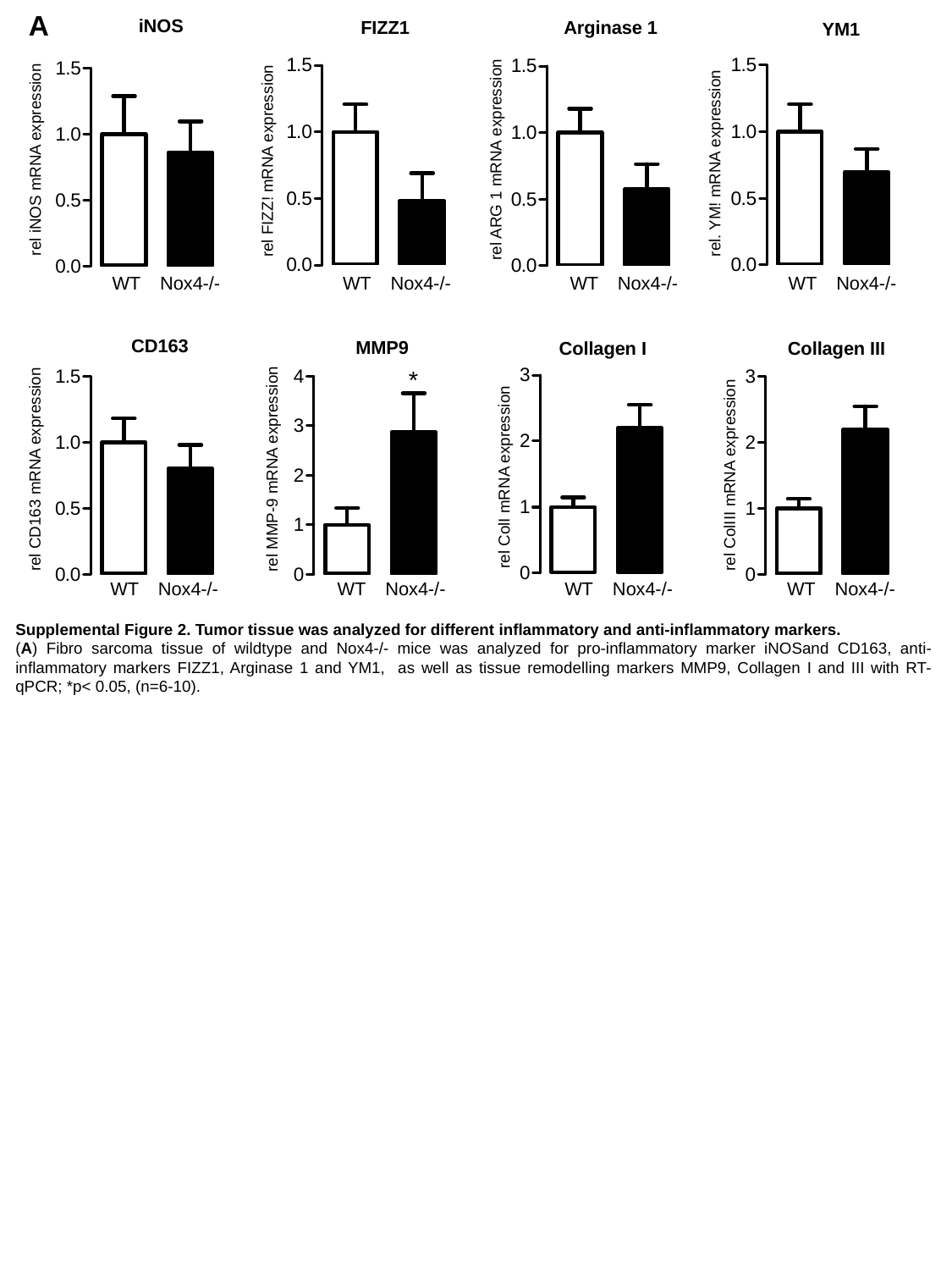

A
iNOS
Arginase 1
FIZZ1
YM1
rel ARG 1 mRNA expression
rel iNOS mRNA expression
rel FIZZ! mRNA expression
rel. YM! mRNA expression
WT
Nox4-/-
WT
Nox4-/-
WT
Nox4-/-
WT
Nox4-/-
CD163
MMP9
Collagen III
Collagen I
*
rel MMP-9 mRNA expression
rel CD163 mRNA expression
rel ColIII mRNA expression
rel ColI mRNA expression
WT
Nox4-/-
WT
Nox4-/-
WT
Nox4-/-
WT
Nox4-/-
Supplemental Figure 2. Tumor tissue was analyzed for different inflammatory and anti-inflammatory markers.
(A) Fibro sarcoma tissue of wildtype and Nox4-/- mice was analyzed for pro-inflammatory marker iNOSand CD163, anti-inflammatory markers FIZZ1, Arginase 1 and YM1, as well as tissue remodelling markers MMP9, Collagen I and III with RT-qPCR; *p< 0.05, (n=6-10).

## Slide 3
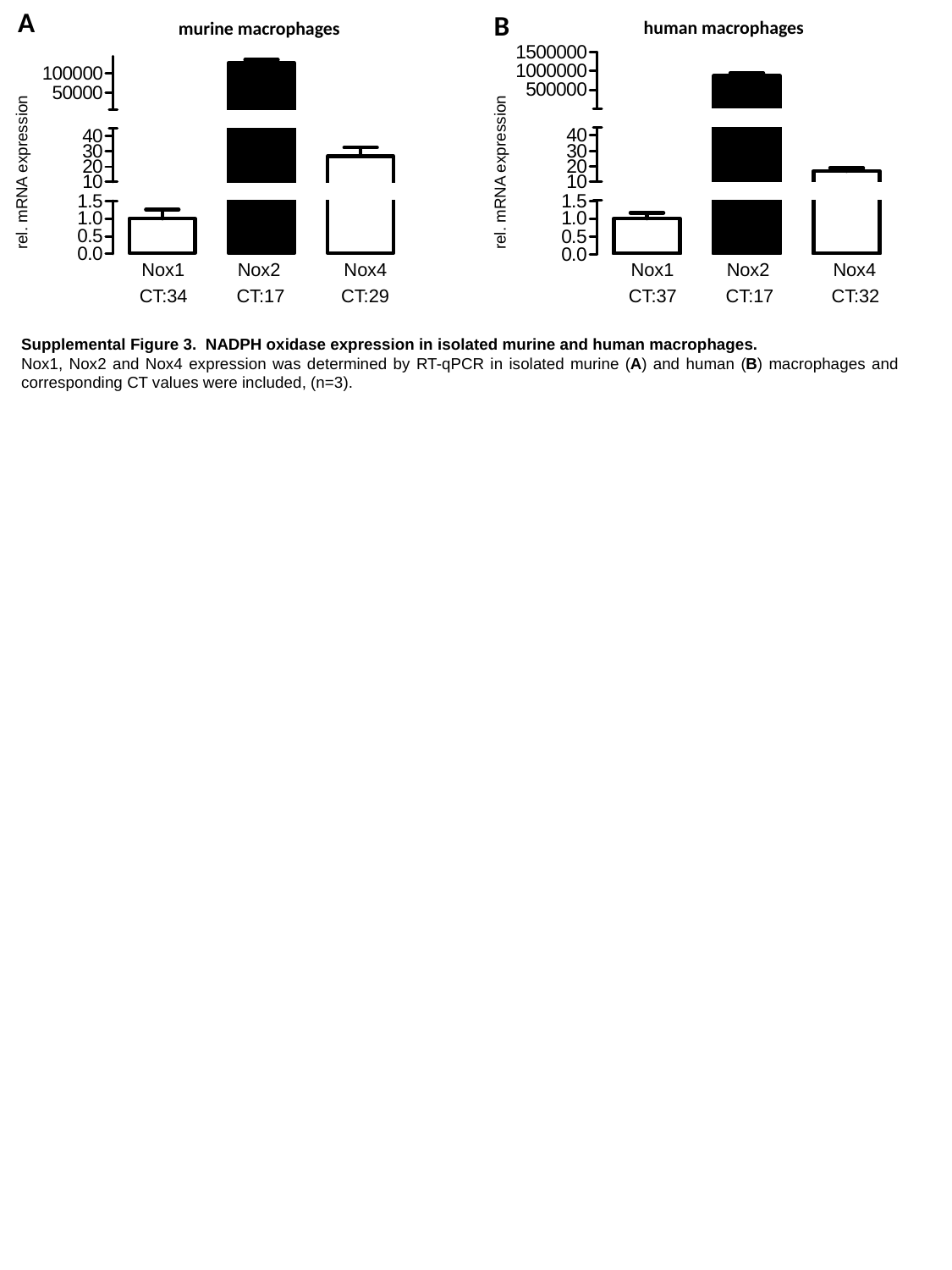

A
B
human macrophages
murine macrophages
rel. mRNA expression
rel. mRNA expression
Nox1
Nox2
Nox4
Nox1
Nox2
Nox4
CT:34
CT:17
CT:29
CT:37
CT:17
CT:32
Supplemental Figure 3. NADPH oxidase expression in isolated murine and human macrophages.
Nox1, Nox2 and Nox4 expression was determined by RT-qPCR in isolated murine (A) and human (B) macrophages and corresponding CT values were included, (n=3).

## Slide 4
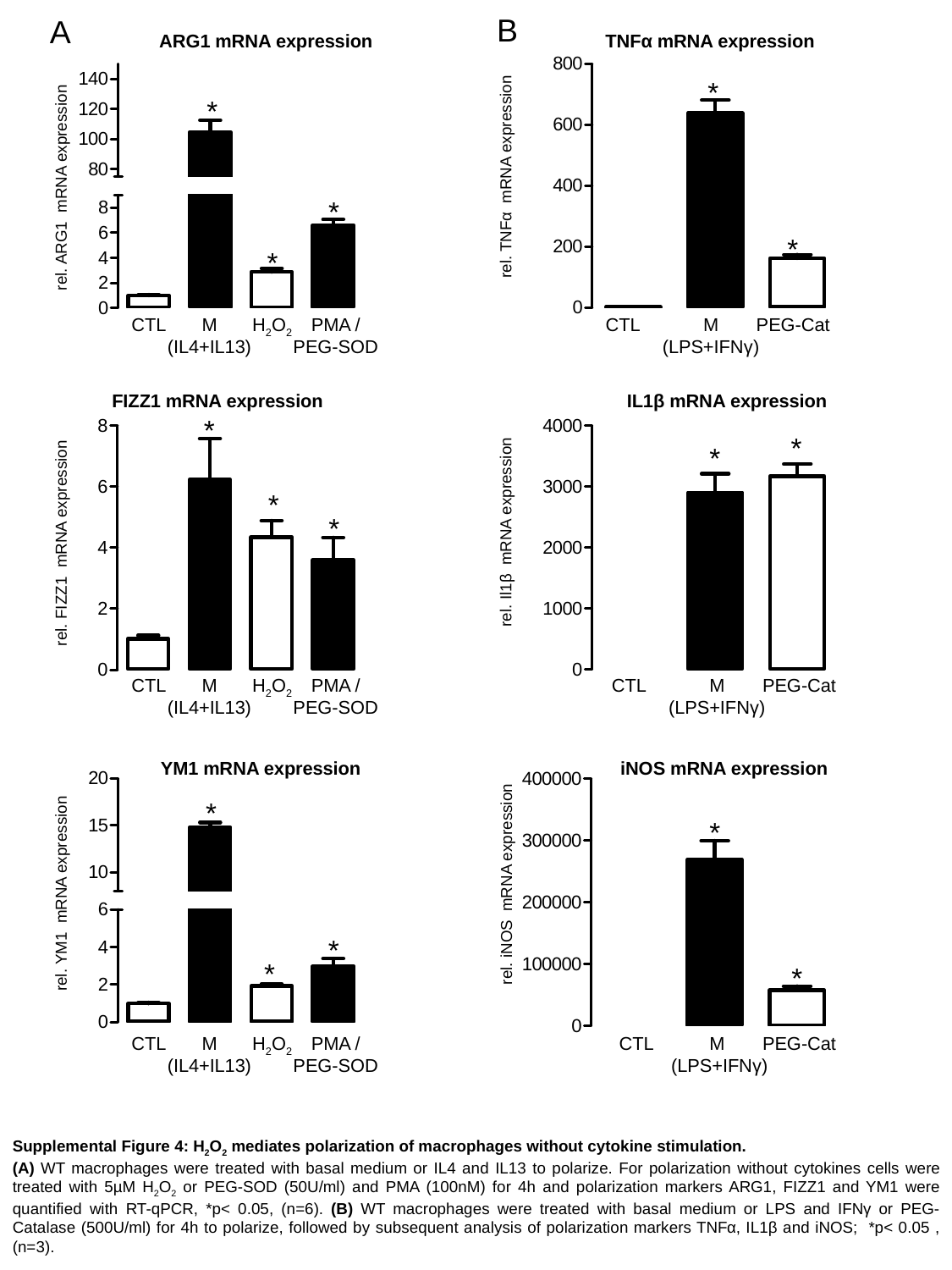

B
A
ARG1 mRNA expression
TNFα mRNA expression
*
*
*
*
*
rel. TNFα mRNA expression
rel. ARG1 mRNA expression
CTL
M
(IL4+IL13)
H2O2
PMA /
PEG-SOD
CTL
M
(LPS+IFNγ)
PEG-Cat
FIZZ1 mRNA expression
IL1β mRNA expression
*
*
*
*
*
rel. Il1β mRNA expression
rel. FIZZ1 mRNA expression
CTL
M
(IL4+IL13)
H2O2
PMA /
PEG-SOD
CTL
M
(LPS+IFNγ)
PEG-Cat
*
*
*
*
*
YM1 mRNA expression
iNOS mRNA expression
rel. iNOS mRNA expression
rel. YM1 mRNA expression
CTL
M
(IL4+IL13)
H2O2
PMA /
PEG-SOD
CTL
M
 (LPS+IFNγ)
PEG-Cat
Supplemental Figure 4: H2O2 mediates polarization of macrophages without cytokine stimulation.
(A) WT macrophages were treated with basal medium or IL4 and IL13 to polarize. For polarization without cytokines cells were treated with 5µM H2O2 or PEG-SOD (50U/ml) and PMA (100nM) for 4h and polarization markers ARG1, FIZZ1 and YM1 were quantified with RT-qPCR, *p< 0.05, (n=6). (B) WT macrophages were treated with basal medium or LPS and IFNγ or PEG-Catalase (500U/ml) for 4h to polarize, followed by subsequent analysis of polarization markers TNFα, IL1β and iNOS; *p< 0.05 ,(n=3).

## Slide 5
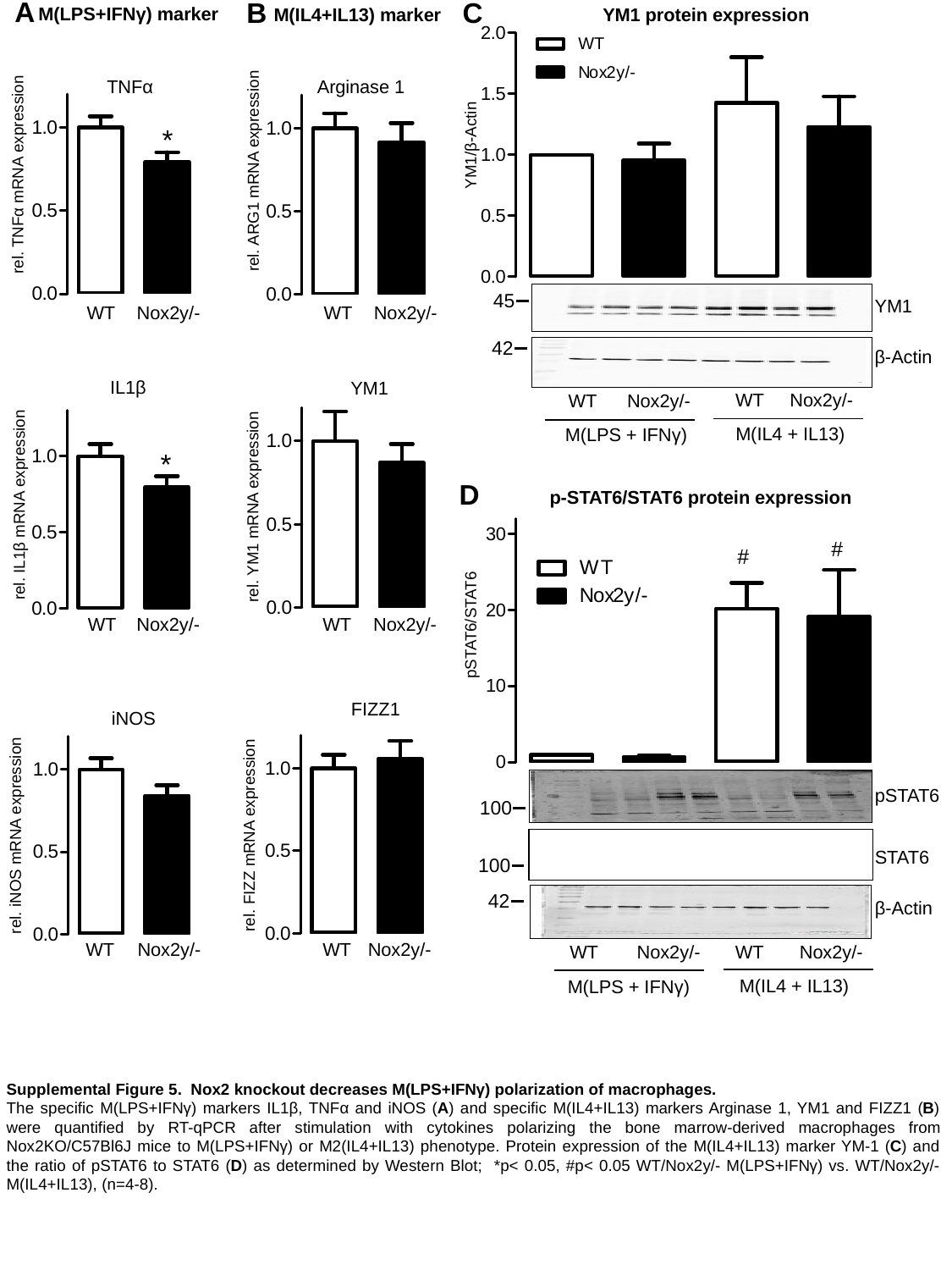

A
B
C
M(LPS+IFNγ) marker
YM1 protein expression
M(IL4+IL13) marker
Arginase 1
TNFα
*
YM1/β-Actin
rel. ARG1 mRNA expression
rel. TNFα mRNA expression
45
YM1
WT
Nox2y/-
WT
Nox2y/-
42
β-Actin
IL1β
YM1
WT
Nox2y/-
WT
Nox2y/-
M(IL4 + IL13)
M(LPS + IFNγ)
*
D
p-STAT6/STAT6 protein expression
rel. IL1β mRNA expression
rel. YM1 mRNA expression
#
#
WT
Nox2y/-
WT
Nox2y/-
pSTAT6/STAT6
FIZZ1
iNOS
pSTAT6
*
100
rel. FIZZ mRNA expression
rel. iNOS mRNA expression
STAT6
100
42
β-Actin
WT
Nox2y/-
WT
Nox2y/-
WT
Nox2y/-
WT
Nox2y/-
M(IL4 + IL13)
M(LPS + IFNγ)
Supplemental Figure 5. Nox2 knockout decreases M(LPS+IFNγ) polarization of macrophages.
The specific M(LPS+IFNγ) markers IL1β, TNFα and iNOS (A) and specific M(IL4+IL13) markers Arginase 1, YM1 and FIZZ1 (B) were quantified by RT-qPCR after stimulation with cytokines polarizing the bone marrow-derived macrophages from Nox2KO/C57Bl6J mice to M(LPS+IFNγ) or M2(IL4+IL13) phenotype. Protein expression of the M(IL4+IL13) marker YM-1 (C) and the ratio of pSTAT6 to STAT6 (D) as determined by Western Blot; *p< 0.05, #p< 0.05 WT/Nox2y/- M(LPS+IFNγ) vs. WT/Nox2y/- M(IL4+IL13), (n=4-8).

## Slide 6
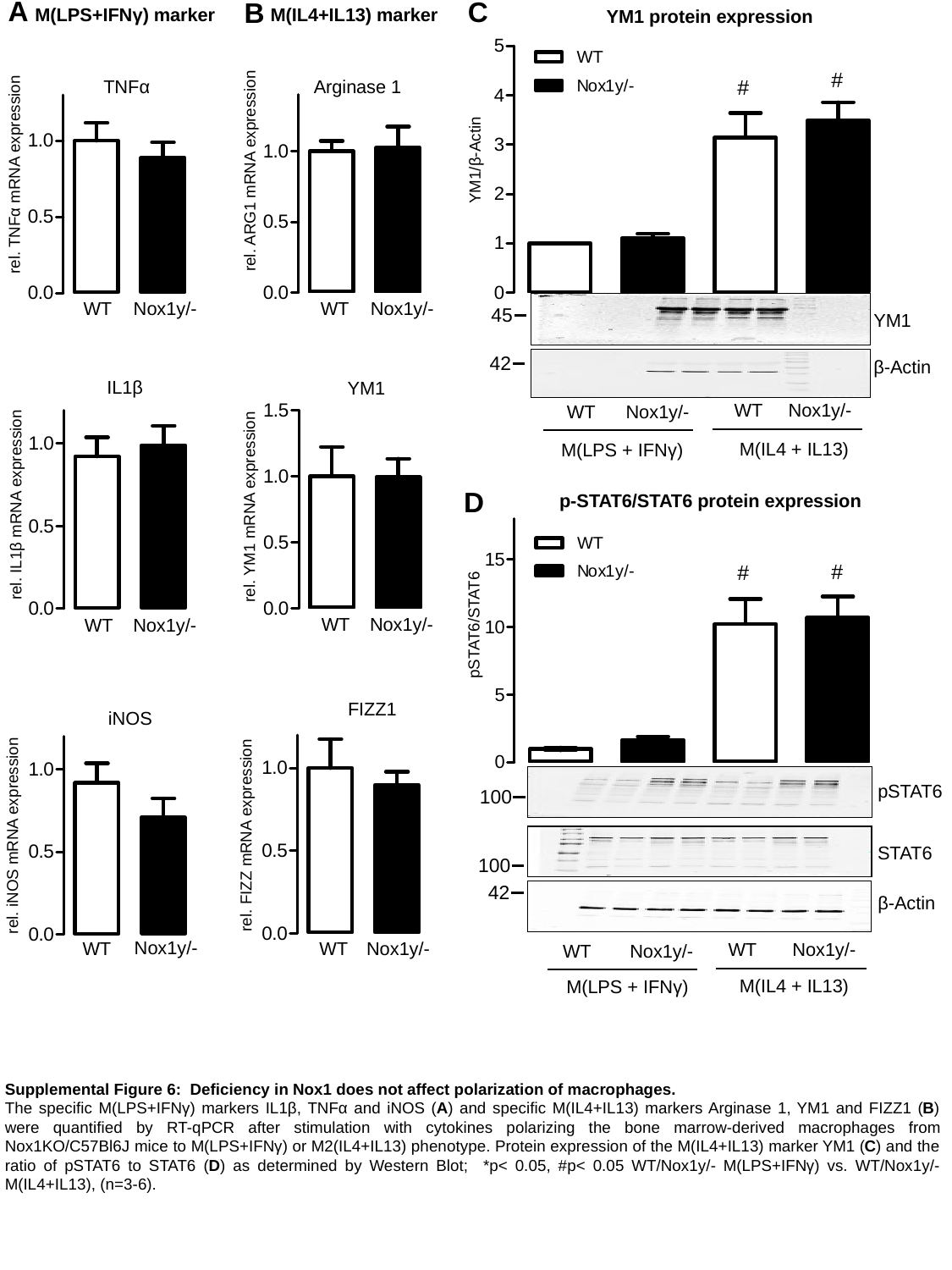

A
C
B
M(IL4+IL13) marker
M(LPS+IFNγ) marker
YM1 protein expression
#
#
Arginase 1
TNFα
YM1/β-Actin
rel. ARG1 mRNA expression
rel. TNFα mRNA expression
WT
Nox1y/-
WT
Nox1y/-
45
YM1
42
β-Actin
IL1β
YM1
WT
Nox1y/-
WT
Nox1y/-
M(IL4 + IL13)
M(LPS + IFNγ)
D
p-STAT6/STAT6 protein expression
rel. IL1β mRNA expression
rel. YM1 mRNA expression
#
#
WT
Nox1y/-
WT
Nox1y/-
pSTAT6/STAT6
FIZZ1
iNOS
pSTAT6
100
rel. FIZZ mRNA expression
rel. iNOS mRNA expression
STAT6
100
42
β-Actin
Nox1y/-
WT
Nox1y/-
WT
WT
Nox1y/-
WT
Nox1y/-
M(IL4 + IL13)
M(LPS + IFNγ)
Supplemental Figure 6: Deficiency in Nox1 does not affect polarization of macrophages.
The specific M(LPS+IFNγ) markers IL1β, TNFα and iNOS (A) and specific M(IL4+IL13) markers Arginase 1, YM1 and FIZZ1 (B) were quantified by RT-qPCR after stimulation with cytokines polarizing the bone marrow-derived macrophages from Nox1KO/C57Bl6J mice to M(LPS+IFNγ) or M2(IL4+IL13) phenotype. Protein expression of the M(IL4+IL13) marker YM1 (C) and the ratio of pSTAT6 to STAT6 (D) as determined by Western Blot; *p< 0.05, #p< 0.05 WT/Nox1y/- M(LPS+IFNγ) vs. WT/Nox1y/- M(IL4+IL13), (n=3-6).

## Slide 7
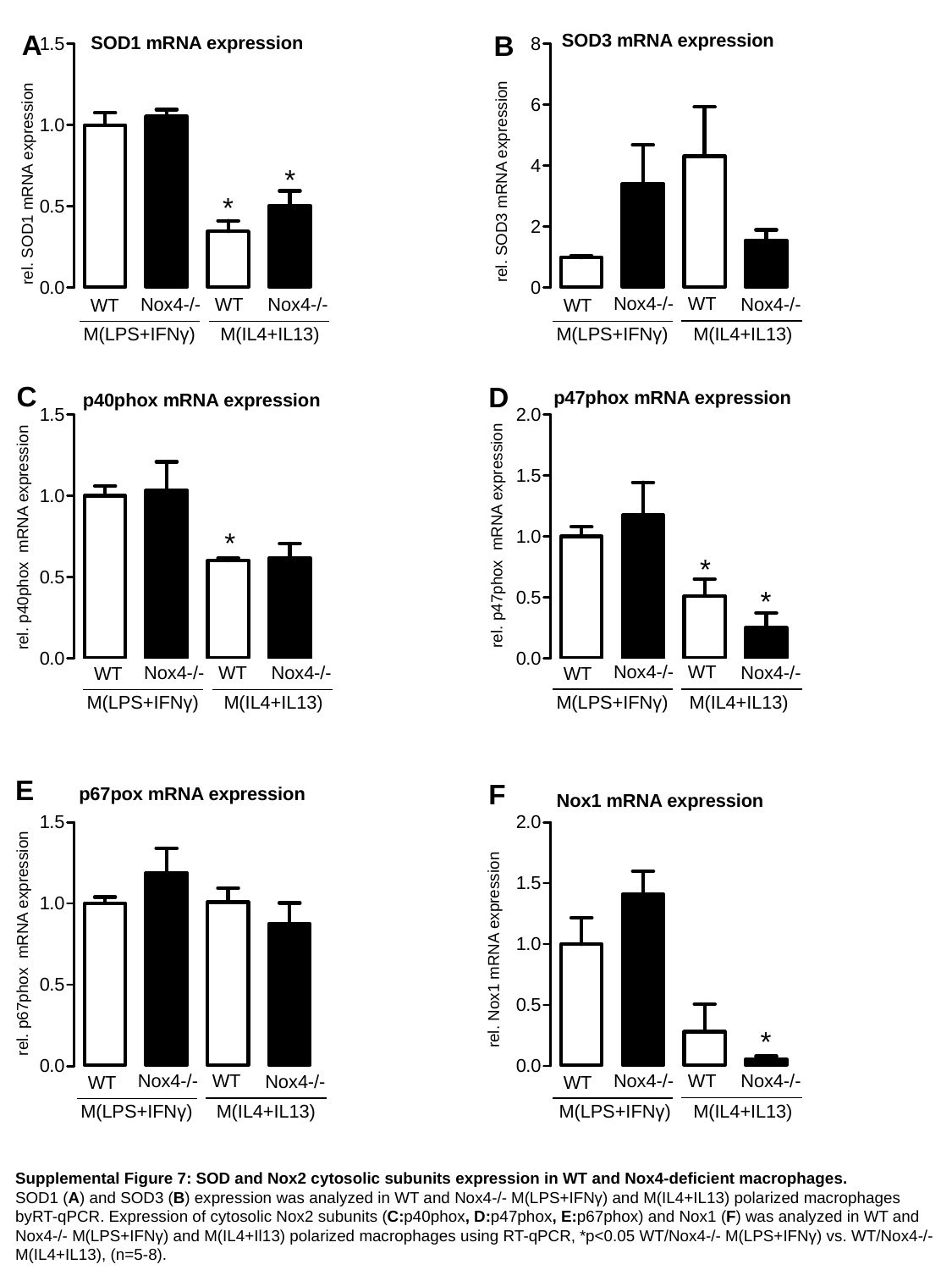

A
B
SOD3 mRNA expression
SOD1 mRNA expression
*
rel. SOD3 mRNA expression
rel. SOD1 mRNA expression
*
Nox4-/-
WT
Nox4-/-
WT
M(LPS+IFNγ)
M(IL4+IL13)
Nox4-/-
WT
Nox4-/-
WT
M(LPS+IFNγ)
M(IL4+IL13)
C
D
p47phox mRNA expression
p40phox mRNA expression
*
rel. p47phox mRNA expression
rel. p40phox mRNA expression
*
*
Nox4-/-
WT
Nox4-/-
WT
M(LPS+IFNγ)
M(IL4+IL13)
Nox4-/-
WT
Nox4-/-
WT
M(LPS+IFNγ)
M(IL4+IL13)
E
F
p67pox mRNA expression
Nox1 mRNA expression
rel. p67phox mRNA expression
rel. Nox1 mRNA expression
*
Nox4-/-
WT
Nox4-/-
WT
M(LPS+IFNγ)
M(IL4+IL13)
Nox4-/-
WT
Nox4-/-
WT
M(LPS+IFNγ)
M(IL4+IL13)
Supplemental Figure 7: SOD and Nox2 cytosolic subunits expression in WT and Nox4-deficient macrophages.
SOD1 (A) and SOD3 (B) expression was analyzed in WT and Nox4-/- M(LPS+IFNγ) and M(IL4+IL13) polarized macrophages byRT-qPCR. Expression of cytosolic Nox2 subunits (C:p40phox, D:p47phox, E:p67phox) and Nox1 (F) was analyzed in WT and Nox4-/- M(LPS+IFNγ) and M(IL4+Il13) polarized macrophages using RT-qPCR, *p<0.05 WT/Nox4-/- M(LPS+IFNγ) vs. WT/Nox4-/- M(IL4+IL13), (n=5-8).

## Slide 8
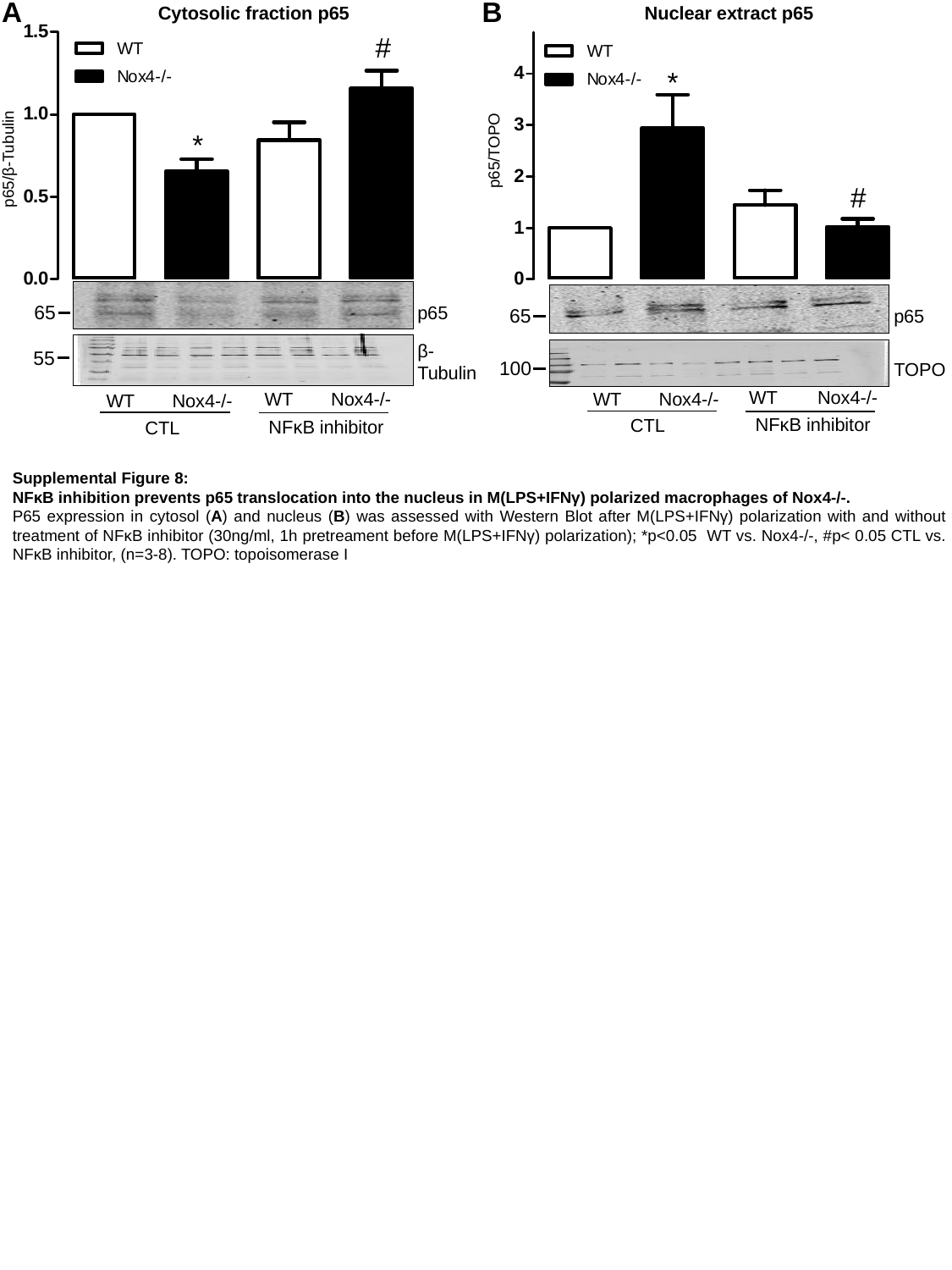

A
B
Cytosolic fraction p65
Nuclear extract p65
#
*
*
p65/TOPO
p65/β-Tubulin
#
65
p65
65
p65
β-
Tubulin
55
100
TOPO
WT
Nox4-/-
WT
Nox4-/-
WT
Nox4-/-
WT
Nox4-/-
NFκB inhibitor
CTL
NFκB inhibitor
CTL
Supplemental Figure 8:
NFκB inhibition prevents p65 translocation into the nucleus in M(LPS+IFNγ) polarized macrophages of Nox4-/-.
P65 expression in cytosol (A) and nucleus (B) was assessed with Western Blot after M(LPS+IFNγ) polarization with and without treatment of NFκB inhibitor (30ng/ml, 1h pretreament before M(LPS+IFNγ) polarization); *p<0.05 WT vs. Nox4-/-, #p< 0.05 CTL vs. NFκB inhibitor, (n=3-8). TOPO: topoisomerase I

## Slide 9
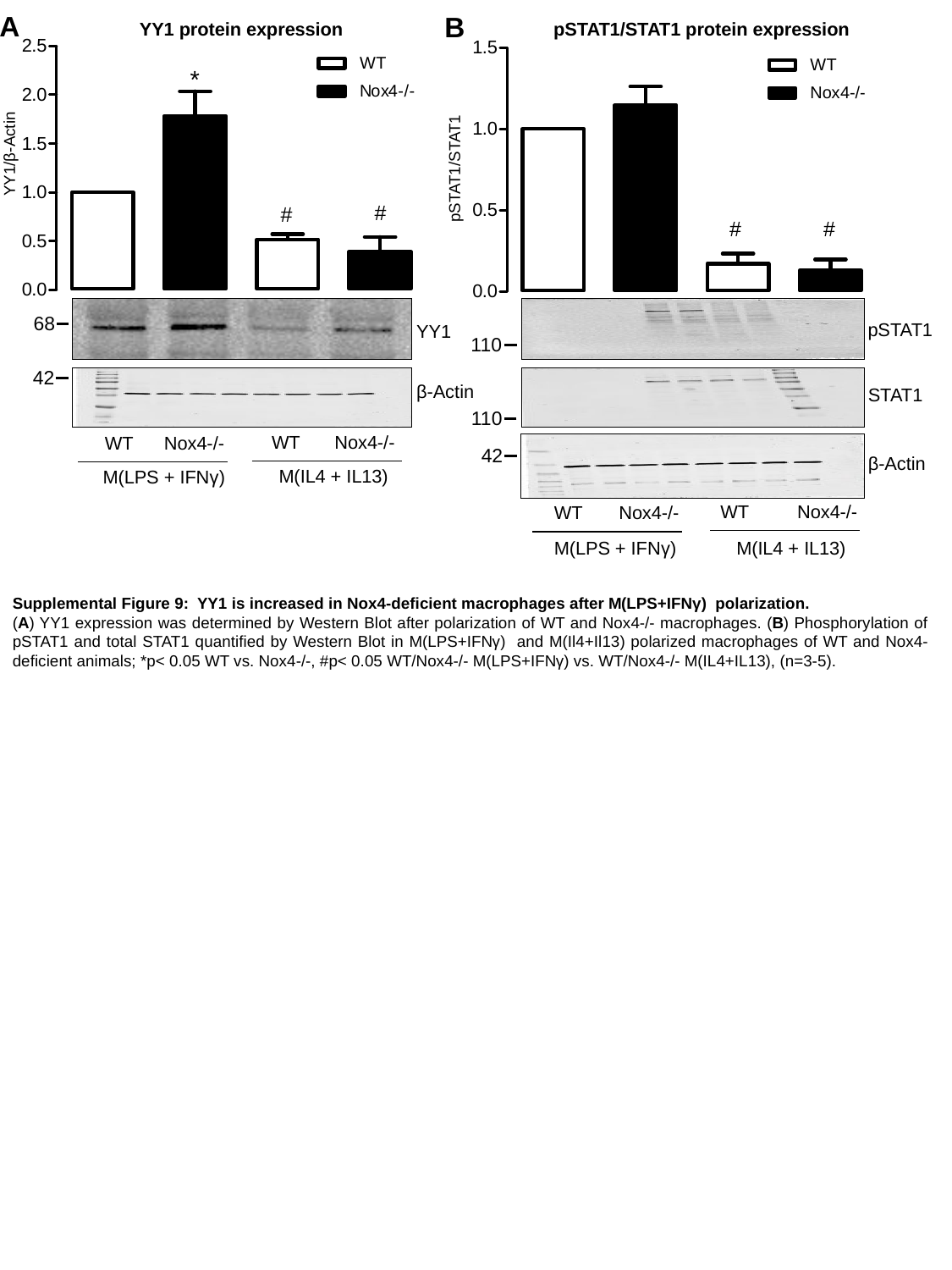

A
B
pSTAT1/STAT1 protein expression
YY1 protein expression
*
YY1/β-Actin
pSTAT1/STAT1
#
#
#
#
68
pSTAT1
YY1
110
42
β-Actin
STAT1
110
WT
Nox4-/-
WT
Nox4-/-
42
β-Actin
M(IL4 + IL13)
M(LPS + IFNγ)
WT
Nox4-/-
WT
Nox4-/-
M(LPS + IFNγ)
M(IL4 + IL13)
Supplemental Figure 9: YY1 is increased in Nox4-deficient macrophages after M(LPS+IFNγ) polarization.
(A) YY1 expression was determined by Western Blot after polarization of WT and Nox4-/- macrophages. (B) Phosphorylation of pSTAT1 and total STAT1 quantified by Western Blot in M(LPS+IFNγ) and M(Il4+Il13) polarized macrophages of WT and Nox4-deficient animals; *p< 0.05 WT vs. Nox4-/-, #p< 0.05 WT/Nox4-/- M(LPS+IFNγ) vs. WT/Nox4-/- M(IL4+IL13), (n=3-5).

## Slide 10
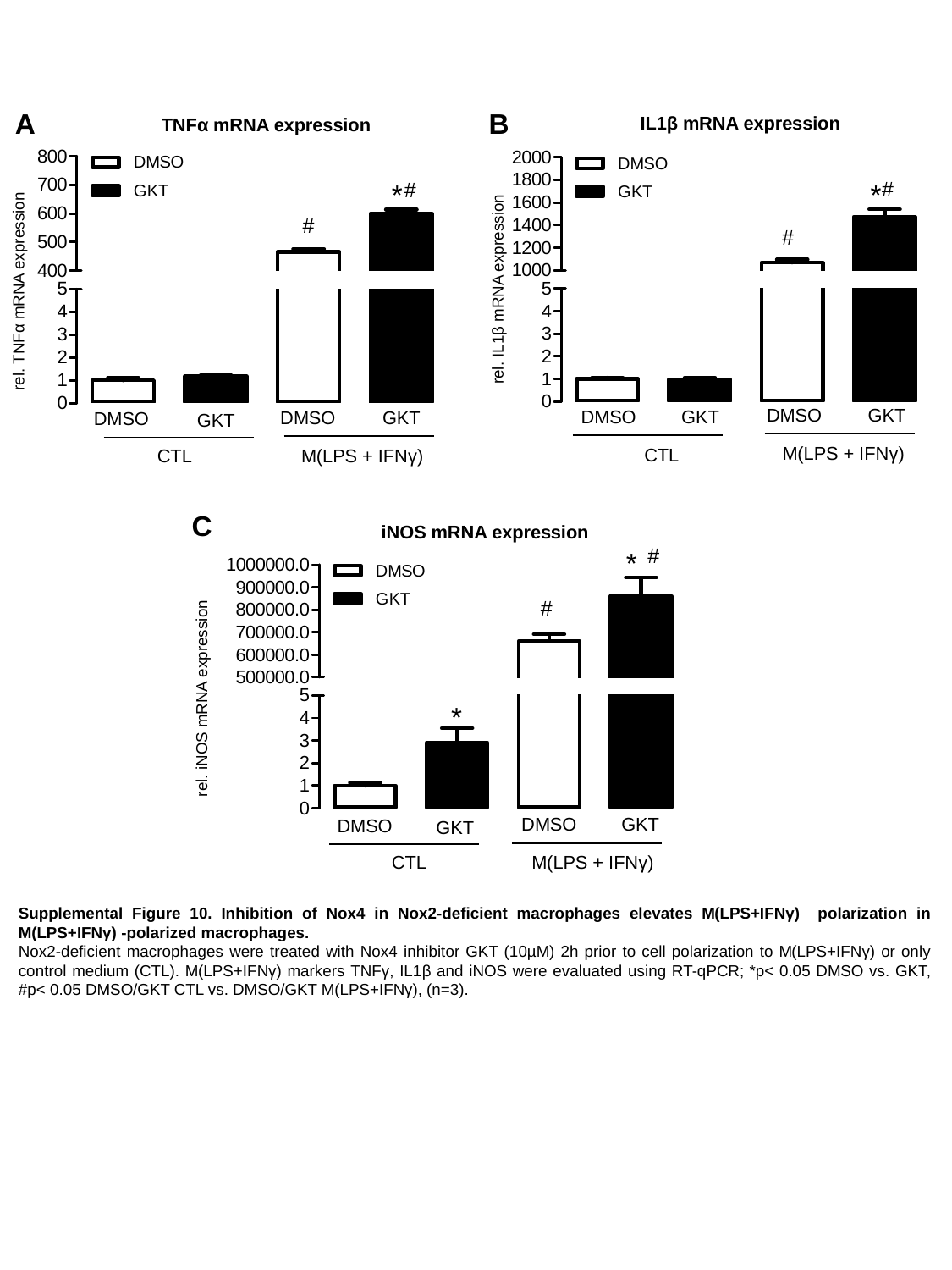

A
B
IL1β mRNA expression
TNFα mRNA expression
#
#
*
*
#
#
rel. IL1β mRNA expression
rel. TNFα mRNA expression
DMSO
GKT
DMSO
GKT
DMSO
GKT
DMSO
GKT
M(LPS + IFNγ)
CTL
CTL
M(LPS + IFNγ)
C
iNOS mRNA expression
#
*
#
rel. iNOS mRNA expression
*
DMSO
GKT
DMSO
GKT
CTL
M(LPS + IFNγ)
Supplemental Figure 10. Inhibition of Nox4 in Nox2-deficient macrophages elevates M(LPS+IFNγ) polarization in M(LPS+IFNγ) -polarized macrophages.
Nox2-deficient macrophages were treated with Nox4 inhibitor GKT (10µM) 2h prior to cell polarization to M(LPS+IFNγ) or only control medium (CTL). M(LPS+IFNγ) markers TNFγ, IL1β and iNOS were evaluated using RT-qPCR; *p< 0.05 DMSO vs. GKT, #p< 0.05 DMSO/GKT CTL vs. DMSO/GKT M(LPS+IFNγ), (n=3).
